# New light on names and naming of dark taxa

**DOI:** 10.3897/mycokeys.30.24376

**Published:** 2018-02-23

**Authors:** Martin Ryberg, R. Henrik Nilsson

**Affiliations:** 1 Department of Organismal Biology, Evolutionary Biology Centre, Uppsala University, Norbyvägen 18D, 752 36 Uppsala, Sweden; 2 Department of Biological and Environmental Sciences, University of Gothenburg, Box 463, 405 30 Göteborg, Sweden; 3 Gothenburg Global Biodiversity Centre, Box 461, 405 30 Göteborg, Sweden

**Keywords:** Taxonomy, nomenclature, mycology, biodiversity

## Abstract

A growing proportion of fungal species and lineages are known only from sequence data and cannot be linked to any physical specimen or resolved taxonomic name. Such fungi are often referred to as “dark taxa” or “dark matter fungi”. As they lack a taxonomic identity in the form of a name, they are regularly ignored in many important contexts, for example in legalisation and species counts. It is therefore very urgent to find a system to also deal with these fungi. Here, issues relating to the taxonomy and nomenclature of dark taxa are discussed and a number of questions that the mycological community needs to consider before deciding on what system/s to implement are highlighted.

## Introduction

*The first step in wisdom is to know the things themselves; this notion consists in having a true idea of the objects; objects are distinguished and known by classifying them methodically and giving them appropriate names. Therefore, classification and name-giving will be the foundation of our science*.

Linnaeus (1735)

Public DNA sequence databases abound with fungal entries that defy all attempts at taxonomic identification. These poorly understood lineages are known from more or less all imaginable substrates and environments, including soil, wood and water but also spacecraft, tumuli and residential areas (e.g. Nilsson et al. 2016). They have been referred to as “dark taxa” or “dark matter fungi” (Page 2016; Grossart et al. 2016) and most of them likely represent undescribed taxa (Tedersoo and Smith 2017), while a limited proportion presumably represents described, but never before sequenced, taxa (cf. Nagy et al. 2011). The number of dark taxa is growing rapidly in the wake of the increasing use of sequence-based approaches to characterisation of biodiversity. Today, few researchers would contest that dark taxa merit both scientific and societal interest, and there is a growing need to classify and communicate these taxa and to record and accumulate data on them. Biodiversity data collections and legislation, in sharp contrast, are largely centred on names of species and higher groups. Being nameless, the dark taxa are not easily incorporated into many of these contexts and are consequently often omitted. They are, for example, usually left out of species counts of areas and therefore from decisions on nature conservation. This unsatisfactory situation where much of our resources and infrastructure cannot properly accommodate dark taxa has spurred a debate in the life sciences on how they should be handled (Samyn and De Clerck 2012; Patterson 2014).

The mycological community has been aware of the problem of dark taxa for a long time (Nilsson et al. 2005; Porter et al. 2008; Ryberg et al. 2008; Hibbett et al. 2009) and several solutions have been proposed. The UNITE database for molecular identification of fungi, for instance, has presented a system where ITS sequence clusters are presented as species hypotheses tagged with unique digital object identifiers (DOIs) to facilitate unambiguous communication (Kõljalg et al. 2016), while Hawksworth et al. (2016) suggested that it should be possible to publish valid names under the International Code of Nomenclature for algae, fungi and plants (ICN) using a DNA sequence, instead of a physical specimen, as type. However, there is no consensus on how the dark taxa should be handled and mycology clearly faces a number of extremely difficult questions in the very near future. These questions will dictate how we refer to, name and to some extent identify, fungi – in short, how mycology is done. The pivotal nature of the pending decisions suggests that all perspectives and points of view must be brought to the surface and vetted thoroughly. Yet it is felt, when following the debate, that this has not been the case so far. In this opinion piece, the authors wish to clarify several overlooked and perhaps obscured aspects of dark taxa and their naming. While a stand will not be taken regarding whether DNA sequences should suffice as species types, it is hoped that several matters that should be resolved will be identified before such a decision can be made. This commentary’s contribution is meant for mycologists at large, because dark taxa concern mycology at large. Through longstanding work with difficult-to-identify fungal lineages from soil and other environmental samples, the authors can attest to the frequency and the widespread nature of dark taxa across the fungal tree of life. Disregarding these un-named taxa for the simple reason that they lack a formal name would be a severe, costly mistake – and one that has already impeded mycology for far too long. This is an issue whose resolution can no longer be postponed.

## Nomenclature and taxonomy

In the context of dark taxa, the distinction between taxonomy (delimiting and characterising taxa) and nomenclature (naming and, to some extent, diagnosing taxa) must be stressed. While these are connected and commonly discussed together, blurring their distinction, they are fundamentally different (de Queiroz 2006). The ICN only governs taxon names and the process of naming taxa; it is by design essentially silent on the processes of characterising and delimiting taxa. A name can therefore be the correct name for a taxon according to the code, even if the taxon delimitation/characterisation with which it was published does not suit the taxon. In analogy, a taxon description can be suitable for a taxon even if the name with which it is published is not the correct name or even a validly published name.

According to the ICN, only a validly published name can be considered for the correct name of a taxon (the name that should be used for the taxon). Two of the requirements for a name to be validly published are that it is published with a diagnosis or description and that a type is designated. The diagnosis can be based on molecular characters (Tripp and Lendemer 2014; Sheikh et al. 2017), while the type of a species name needs to be a physical specimen or exceptionally, an illustration of a specimen. Names of taxa of higher rank have a taxon at the immediate lower rank as their type and thus also refer back to a type specimen in the end. The type indicates what taxon a name refers to, and all validly published names whose type is included in the taxon should be considered for that taxon. Determining which name goes where is sometimes made more complicated by the fact that types may be difficult to get hold of or may even be missing altogether for some names. In other cases, the type may not manifest all characters needed to clarify to which taxon it belongs. When the type is missing or lacking important characters, new or additional types can be designated (neo- and epi-types, respectively; Ariyawansa et al. 2014). The basic principle to establish the correct name for a taxon is that the first validly published name should be used, although there are exceptions to this rule. Given that all validly published names must be considered for a taxon, an inflation in names that are difficult to interpret or apply may hamper taxonomy more than an inflation in species descriptions without valid names.

Since the description of taxa is only controlled by the regular scientific principles for publishing, taxa may be described and diagnosed based solely on molecular characters. Such taxa can also be given a name (De Beer et al. 2016), although such names cannot be considered validly published unless there is a specimen to serve as type. This is what the Hawksworth et al. (2016) proposal seeks to amend. The issue with dark taxa is thus not that they cannot be described, but that they cannot be given formal names for communication.

## What are names for?

Names are used for communicating objects and concepts. Unless an object or concept is very straightforward indeed, the lack of a name is a major obstacle in its communication and may – implicitly or not – be taken to mean that its communication is not necessary to begin with. This is a general societal issue, but it certainly pertains to mycology as well. Many newly described taxa have, in fact, been represented in DNA sequence databases for 10 years or more before they piqued somebody’s interest or were possible to typify according to the ICN (see examples in Nilsson et al. 2016). Upon closer inspection, several of these taxa were found to be both ubiquitous and of significant taxonomic and ecological interest (Rosling et al. 2011; James and Seifert 2017). There is, thus, data to suggest both that the lack of names for dark taxa have retarded progress on their study and that there is ample reason to study and communicate dark taxa in the first place.

Communication of taxa includes aspects such as incorporation into biodiversity datasets, sequence repositories and legislation but also regular scientific and societal communication. What type of name to use will depend on the identity of the communicating parties. For computers, accession numbers such as DOIs will suffice for communication. DOIs are, however, less suitable for human communication (e.g. http://dx.doi.org/10.15156/BIO/SH004915.07FU versus *Vishniacozyma victoriae*). For scientists, latinised binomials may work well, while society at large may prefer vernacular names.

For efficient communication, it is important to consider that one taxon should have only one name and that any name should refer to only one taxon. One of the major purposes of the ICN is to ensure and uphold these relations, while vernacular names are not governed by and, indeed, often violate such rules. The use of parallel naming systems does little to facilitate unambiguous naming in biological systematics. For instance, one and the same name can be the correct or valid name for different taxa under the ICN and the International Code for Zoological nomenclature at the same time, e.g. *Erica* in Ericaceae (Viridiplantae) and Arachnida (Metazoa). For ambiregnal taxa, different names may be the correct/valid name under different nomenclature codes. For instance, a dinoflagellate genus was named *Phalacroma* under the ICN, a name that subsequently was found to be already occupied under the zoological code. Thus, the name *Prodinophysis* was introduced for the same genus (Taylor et al. 1987). In the case where different names are used for the same taxon, databases can link the different uses of names across the systems.

## Delimitation of taxa

Descriptions of taxa may be based on different sets of characters, for example sexual or asexual reproductive structures, physiological parameters or DNA bases. It may therefore be difficult to tell whether a taxon, described based on one type of character, is the same as a taxon described from another character type or set of characters. This is the basis behind both the former dual system of naming for “Eumycota” vis-à-vis “Deuteromycota” and the situation which is now faced with dark taxa. In the case of dark taxa, it is not immediately clear how to correlate a species delimited from environmental sequence data to, say, a range of physiological parameters quantified in the lab or a handful of morphological traits gleaned from microscopy studies of soil samples. Obtaining such additional data and mapping them to individual species will not be straightforward from heterogeneous, mixed-species substrates such as soil and water, but emerging single-cell techniques (e.g. Castelle et al. 2015) offer promise in this regard. As overlapping character sets gradually become available, improved understanding of the underlying taxon will follow piece by piece and the correct name can eventually be assigned according to the nomenclature code. In the context of “Eumycota” and “Deuteromycota”, molecular data are often used to link the teleomorph and anamorph stages of species, thus resolving the issue (e.g. Piątek et al. 2017). This is also the reason why sequence data from type specimens are very valuable to sort out nomenclature and DNA barcoding issues and to bring dark taxa under the realm of taxonomy by providing them with a name (Robbertse et al. 2017; Torres-Cruz et al. 2017).

As taxon delimitation and naming are two different things, another complication in what a name refers to is that different taxonomists may advocate different circumscriptions of taxa while the name itself is determined by the ICN. In these cases, the same name may be the correct name for different taxa, with little overlap in the underlying organisms. Furthermore, a name can be correct for some taxon or a synonym of another name depending on the specific taxonomy. If it is required that taxa be monophyletic, then changes in the taxonomy should be expected due to changes in the understanding of evolutionary relationships. Even if this stabilises with time as better estimates of evolutionary relationships are obtained, there may still be conflicts as to what clades are considered as taxa and at what taxonomic level. For example, Hibbett et al. (2007) treat Monoblepharidomycetes as a class in Chytridiomycota while Powell and Letcher (2014) classify it in the monotypic phylum Monoblepharidomycota. Similarly, the small genus *Entorrhiza* is variously recognised as a basidiomycete lineage or as a separate phylum depending on what resource is turned to (Bauer et al. 2015).

The species level is often viewed as a separate evolutionary lineage of special standing (Mayden 1997; but see, e.g. Baum 2009), but there will still be disagreement on how species are delimited. Any species delimitation will always be a hypothesis and different lines of evidence may disagree as to which hypothesis is best supported. Although molecular data provide significant explanatory power in systematics and taxonomy, their use is not devoid of complications. Clustering of sequences into operational taxonomic units, for instance, depends on the choice of clustering algorithm and the parameter settings used (e.g. selection of sequence similarity cut-off) as well as the choice of genetic marker and the individual sequences to be clustered. Thus, equating a sequence-derived operational taxonomic unit with a species is problematic (Schoch et al. 2012; Ryberg 2015). A sequence-derived operational taxonomic unit may, nevertheless, be a species hypothesis.

Without a reference to which taxonomy is employed, what is referred to by a name is more or less ambiguous. The UNITE species hypothesis system provides an unambiguous way to refer to sets of sequences at approximately the species level and additions and removals of sequences to those species hypotheses can be traced back in time (e.g. https://unite.ut.ee/bl_forw_sh.php?sh_name=SH181628.07FU). However, this approach is limited to sequences included in the underlying dataset, the given set of hypotheses and to taxa represented by ITS sequence data in the first place. Changes between taxonomies are a part of the progress in understanding nature and only scientific advances, together with a dialogue amongst scientists to arrive at a consensus, can resolve this problem. However, without some sort of names for communication such progress seems difficult.

## Outlook

The number of dark taxa increases with more or less every new metabarcoding study, but the pace at which these taxa are formalised is many orders of magnitude slower (James and Seifert 2017). This hints at an untenable situation and it is becoming increasingly clear that a system to handle dark taxa in the context of taxonomy, nomenclature and biodiversity at large is needed. The authors plea for the adoption of dark taxa into regular mycology and argue for an expedient establishment of a system or approach to handle dark taxa in mycology and elsewhere. When constructing such a system, many urgent questions present themselves. What are being communicated: sequence clusters, taxa at an undefined level or taxa as recognised by the ICN? Who are communicating: is it computers, scientists, the society at large or any combination of these? If a system with computer-facilitated communication on sequence clusters is wanted, UNITE already fills this role. If a system with a flexible set of taxonomic hypotheses that consider more than just ITS sequence distances is also wanted, something more is needed. For such a system, the impact on biodiversity research should also be considered. Will it encourage and/or deter research and, if so, what kind of research? Should it, unlike the traditional nomenclature codes, encourage best practices in taxonomy? If so, will it achieve these best practices or will it engender poor practices and increased confusion? There is clearly a risk that allowing sequences as types will inflate the number of (rogue?) names and serve to hamper taxonomy in the end (cf. Seifert 2018). As sequences are fundamentally different from a physical specimen, consideration should also be given as to how well sequences will serve as types. It is true that they are perfectly well amenable to digitalisation and that they are easy to share and compare. At the same time, they contain little additional information if further taxonomic resolution is needed and may increase the need for epitypes. Finally, it should be asked if the aim is a separate, DNA-based system for the dark taxa or an integrated system including other characters and taxa too. If the aim is comparable units and not the creation of disjunct taxonomic systems, one system is certainly recommended.

These questions are urgent, because dark taxa permeate mycology and the fungal tree of life, and *ad hoc* names are being used to communicate them without any system to ensure stability of those names (De Beer et al. 2016; Tedersoo and Smith 2017). The answers to the above questions are not immediately clear and they may furthermore differ depending on personal perspectives. A heated debate is therefore expected, perhaps without any consensus at the end. Whatever system is implemented, there needs to be an active discussion in the mycological community as to what confidence should be required for the named taxa and whether a specific system should be implemented to safeguard these quality measures (cf. Tedersoo et al. 2015).

Seifert (2018) asks what is the point of a name when there is no additional information attached to it. At the same time, he gives a species the name “the brain fungus” (not valid according to the ICN) to be able to talk about it and makes a plea for more information on it. However, without a stable and precise name as an identifier, it will be difficult to accumulate precise knowledge about exactly this species. A name is not an end to our understanding of a taxon, but a means and a beginning. If mycology is to be the study of all fungi and not just the perhaps < 10 % which can be readily observed, then dark taxa should be welcomed into the light.
